# Circadian rhythms mediate infection risk in *Daphnia dentifera*

**DOI:** 10.1002/ece3.9264

**Published:** 2022-09-09

**Authors:** Alaina C. Pfenning‐Butterworth, David T. Nguyen, Jessica L. Hite, Clayton E. Cressler

**Affiliations:** ^1^ School of Biological Sciences University of Nebraska‐Lincoln Lincoln Nebraska USA; ^2^ Department of Pathobiological Sciences University of Wisconsin‐Madison Madison Wisconsin USA

**Keywords:** circadian rhythm, *Daphnia dentifera*, feeding behavior, infection, *Metschnikowia bicuspidata*, pathogen exposure

## Abstract

Biological rhythms mediate important within‐host processes such as metabolism, immunity, and behavior which are often linked to combating disease exposure. For many hosts, exposure to pathogens occurs while feeding. However, the link between feeding rhythms and infection risk is unclear because feeding behavior is tightly coupled with immune and metabolic processes which may decrease susceptibility to infection. Here, we use the *Daphnia dentifera–Metschnikowia bicuspidata* host–pathogen system to determine how rhythms in feeding rate and immune function mediate infection risk. The host is known to have a nocturnal circadian rhythm in feeding rate, yet we found that they do not exhibit a circadian rhythm in phenoloxidase activity. We found that the time of day when individuals are exposed to pathogens affects the probability of infection with higher infection prevalence at night, indicating that infection risk is driven by a host's circadian rhythm in feeding behavior. These results suggest that the natural circadian rhythm of the host should be considered when addressing epidemiological dynamics.

## Graphical abstract

The link between biological rhythms in feeding behavior and infection risk is unclear because feeding behavior is tightly coupled with immune and metabolic processes which may affect infection susceptibility. We use the *Daphnia dentifera*–*Metschnikowia bicuspidata* host–pathogen system to determine how rhythms in feeding rate and immune function mediate infection risk. Individuals are more likely to get infected during night exposures, indicating that infection risk is driven by a host's nocturnal circadian rhythm in feeding behavior.

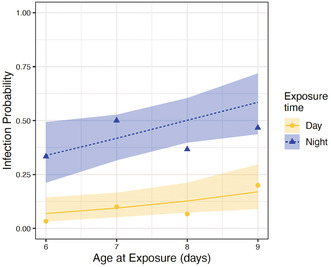

## 1 INTRODUCTION

Biological rhythms enable organisms to coordinate their molecular and physiological processes with the daily and seasonal changes that occur in the environment. Increasing evidence suggests that biological rhythms mediate processes as diverse as feeding behavior, metabolism, and immunity (Li et al., 2020; Scheiermann et al., 2013; Schibler et al., 2003; Serin & Tek, 2019). Circadian rhythms in immunity, in particular, can have obvious implications for infection outcomes. In mice, there is circadian control of TLR9‐mediated immune function and macrophages such that immunity is higher during their active phase, at night (Keller et al., 2009; Silver et al., 2012). Compared with mice infected during the active phase, mice infected during the resting phase had higher burdens of *Salmonella typhimurium*, but lower burdens of *Leishmania major* (Bellet et al., 2013; Kiessling et al., 2017). The difference in response may be due to important ecological or within‐host processes that were not accounted for in the original studies. For example, many nonimmune processes known to exhibit a biological rhythm, for example, feeding behavior, can be mechanistically linked to pathogen exposure, but the impact these rhythms have on the probability or intensity of infection remains unclear.

Resource acquisition (hereafter “feeding behavior”) is important to both the host and the parasite. In many animals, feeding behavior varies over a daily cycle which could introduce daily variation in within‐host processes such as immunity and reproduction. Moreover, many animals are exposed to pathogens while feeding, which could create daily variation in exposure that contributes to daily differences in infection risk.

Here, we use a zooplankton host, *Daphnia dentifera*, to determine how circadian rhythms in feeding behavior and immune function impact infection success. *Daphnia dentifera* are key consumers native to North American freshwater temperate lakes. Circadian rhythms are prominent in the locomotor behaviors that drive diel vertical migration in many species of *Daphnia* wherein individuals migrate toward the water surface at night and return to deep water during the day. Zooplankton diel vertical migration is an ecologically important phenomenon that leads to a massive movement of biomass in both freshwater and marine systems. This pronounced daily movement of individuals from different depths appears to be driven by trade‐offs between reducing predation by visual predators such as fish, damage by ultraviolet radiation, and the advantage of acquiring necessary resources in the warmer surface waters (Haney & Hall, 1975; Leach et al., 2015). Together, these circadian behaviors carry important implications for nutrient cycling, trophic interactions, and disease biology (Haupt et al., 2009, 2010; Overholt et al., 2012).

In addition to the daily locomotor behavior, *D. dentifera* exhibit a circadian rhythm in feeding behavior (Pfenning‐Butterworth et al., 2021), which may directly modulate differences in pathogen exposure and infection outcomes. For the wide array of hosts, including *Daphnia*, that encounter infectious agents while foraging, changes in feeding rates serve as a first line of defense, reducing the infective dose and sequestering resources away from pathogen (Adamo et al., 2010). For example, *Daphnia* are exposed to numerous pathogens while feeding, including the highly virulent and common fungal pathogen, *Metschnikowia bicuspidata*, studied here (Duffy et al., 2010). Because hosts encounter pathogens while foraging, feeding rates are strongly correlated with pathogen exposure rates (Strauss et al., 2019). Not surprisingly then, *D. dentifera* clones with higher feeding rates can suffer higher infection risk with *M. bicuspidata* (Hall et al., 2010; Strauss et al., 2019). A recent study demonstrated that *D. dentifera* feeding rates increase at night relative to the day (Pfenning‐Butterworth et al., 2021). Thus, circadian‐based increases in feeding rates at night could increase exposure and infection rates.

However, increased exposure does not necessarily translate to increased infection because of potential confounding effects of rhythms in immunity affecting susceptibility. Several immune genes are known to exhibit circadian variation in *D. pulex*, including genes involved in pathogen recognition and signal transduction (Rund et al., 2016). Circadian variation in feeding may also influence circadian rhythms in immunity because of the impact of feeding on the accumulation of energy stores required to maintain innate immunity (e.g., triglycerides; Peters, 1987, Buchmann, 2014). In *Daphnia* (and other invertebrates), active phenoloxidase (PO) increases when individuals are exposed to pathogens (Labbe & Little, 2009) and initiates the molecular pathway that produces melanin, which attaches to pathogens to inhibit their growth and replication (Cerenius & Söderhäll, 2004; González‐Santoyo & Córdoba‐Aguilar, 2012; Pauwels et al., 2010; Povey et al., 2014). Many of the genes involved in the activation pathway for PO are rhythmic, with higher activity during the day than at the night (Rund et al., 2016). Additionally, individuals raised on high food levels have higher PO activity (Pauwels et al., 2010). For *Daphnia*, these results suggest that PO might be highest at night when feeding rates are highest. However, given the time required for food intake to be converted to energy, to fuel the immune system, PO may actually peak after the rhythm in feeding. Thus, for hosts that are exposed to pathogens while feeding, determining how circadian rhythms influence the risk of infection requires understanding whether feeding rates and immune function (e.g., PO) are synchronous or asynchronous.

**FIGURE 1:**
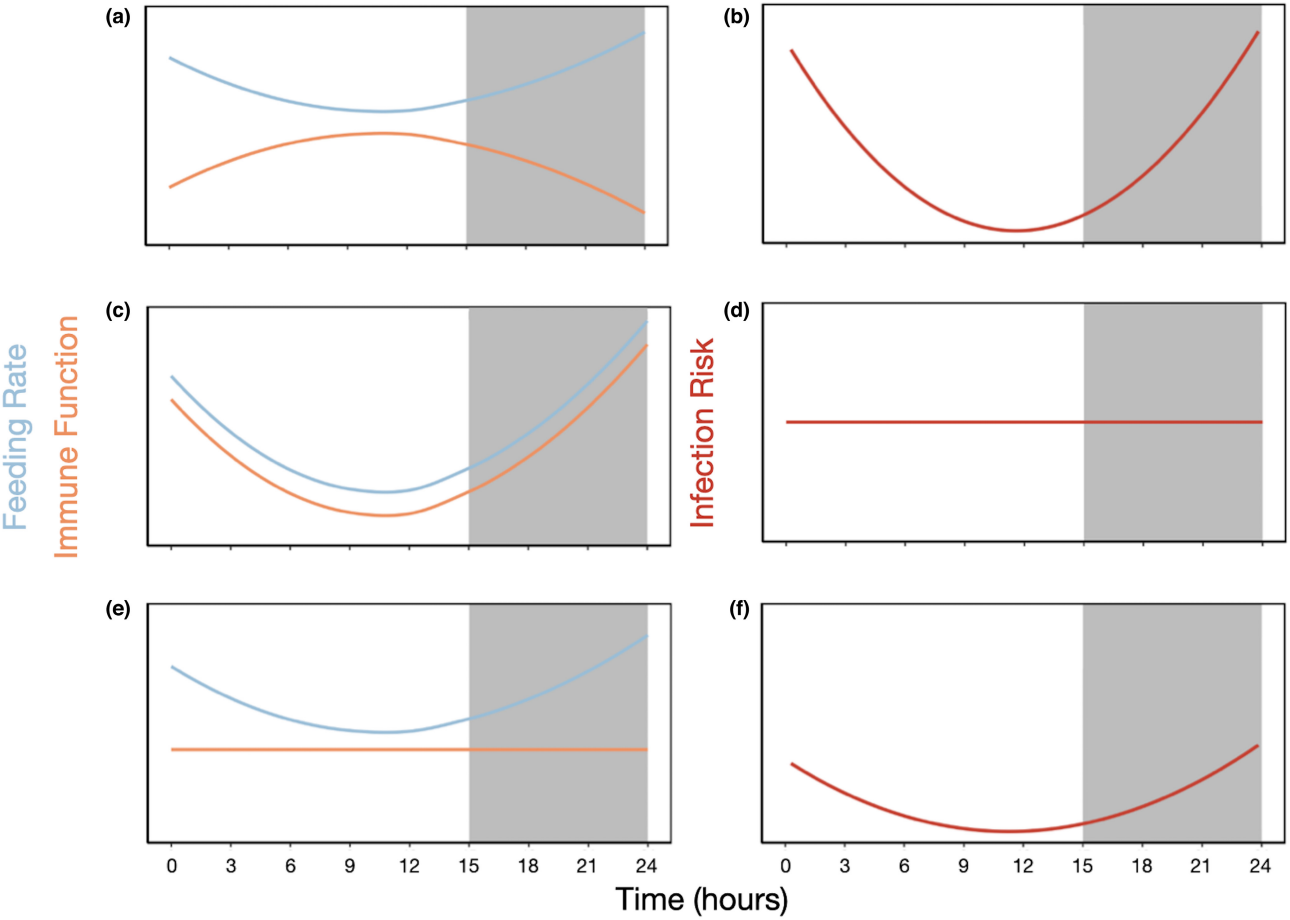
A hypothetical representation of how the presence and direction of a daily rhythm in immune function compared with the daily rhythm in feeding (exposure) could affect infection risk. Plot shading indicates a 15:9 light–dark cycle. (a) When feeding/exposure and immune daily rhythms are out of phase with one another, there would be a daily rhythm in infection risk (b), where infection risk is highest when feeding/exposure is high and immune function is low. (c) When feeding/exposure and immune daily rhythms are in phase with one another, there would be no rhythm in infection risk (d). (e) In the absence of a daily rhythm in immune function, there would be a daily rhythm in infection (f) that is determined by the rhythm in feeding/exposure.

To investigate how circadian rhythms in feeding behavior (pathogen exposure) and immunity interact to affect infection risk, we explored three hypotheses. For all predictions, the circadian rhythm in feeding leads to higher feeding rate during the active phase (night) relative to the resting phase (day; Pfenning‐Butterworth et al., 2021; indicated by the blue line in Figure 1). Hypothesis 1: If rhythms in immune function are out of phase with the feeding rhythm, (i.e., feeding is high when immune function is low, Figure 1a), we predict that infection risk will peak when feeding (exposure) is high and the immune system is least active (Figure 1b). This prediction is supported by documented rhythms in feeding and immune gene expression (Pfenning‐Butterworth et al., 2021; Rund et al., 2016). Hypothesis 2: If rhythms in immune function are in phase with the feeding rhythm, that is, feeding rates and immune function are elevated at the same time (Figure 1c), we predict that infection risk may not show a strong circadian rhythm, because the time of highest exposure is also when the immune system is most active (Figure 1d). This hypothesis is most likely in systems with strong links between feeding and immunity (Pauwels et al., 2010). Hypothesis 3: If rhythms in immune function are absent (Figure 1e), we predict that rhythms in infection risk will mirror rhythms in feeding (exposure) rates. Under this hypothesis, the rhythm in infection risk would appear the same as the first hypothesis, but the amplitude of the rhythm would be smaller because of the lack of a rhythm in immunity (Figure 1f). This hypothesis is most likely when rhythms in precursor gene expression on PO and the effect of the rhythm in feeding rate on PO are counterbalanced. To test these hypotheses, we measured variation in PO activity, feeding rate, and infection outcomes over a daily cycle.

## 2 METHODS

### 2.1 Phenoloxidase assay

To measure circadian‐driven variation in immunity, we used standard methods to differentiate between entrained circadian rhythms and fluctuations driven by light cues. These include measuring PO activity for 3 days under 15:9 light:dark photoperiod and a fourth day in complete darkness to determine whether PO activity patterns persisted without light cues. To reduce any potential maternal effects, experimental neonates were collected from the third clutch of a single genotype of *D. dentifera* originally collected in Southern Michigan (USA) and reared under standard laboratory conditions (55 individuals per 1 L, 15:9 light:dark photoperiod at 22°C) for at least 30 generations. We also took additional precautions to standardize potential variation in laboratory‐reared algal food (*Ankistrodesmus falcatus*) by feeding individuals every other day 1 mgC/L of *A. falcatus* collected from steady‐state chemostat (for details see Hite et al., 2020). This food was used from birth through the duration of the PO assay (which started when *D. dentifera* were 6 days old).

To assess whether *D. dentifera* has a circadian rhythm in phenoloxidase activity (immunity) in the absence of pathogens, we quantified PO activity in the hemolymph (following Mucklow & Ebert, 2003) every 3 h for 4 days (3 days under 15:9 light:dark photoperiod and a fourth day in complete darkness). Hemolymph was collected after pricking individual *D. dentifera* in the heart, while they were still alive (28‐gauge needle, Monject). To obtain enough material for analysis, the hemolymph of eight individuals was pooled to reach a final volume of 2 μl; this was added to 150 μl of PBS buffer on ice (0.15 M NaCl, 10 mM Na_2_HPO_4_.2H_2_0, pH 7.5). Next, 50 μl of the hemolymph‐PBS solution was transferred to 225 μl of 20 mM L‐Dopa (in duplicate). The absorbance at 475 nm was measured immediately and every 30 min for 4.5 h (Tecan©, Maennedorf, Switzerland). Enzyme and PO activity increased linearly during the 4.5 h, indicating that there was no degradation occurring during the assay. We calculated PO activity as the increase in absorbance after 4.5 h (absorbance at 4.5 h – absorbance at 0 h) corrected by changes in the control (PBS and L‐Dopa only). We calculated PO active units as the corrected change over 4.5 h*1000 (following Mucklow & Ebert, 2003) and then divided by the number of individuals in the sample to get a measure of active PO per individual that could be compared against the individual measurements of feeding rate.

We used the BioDare2 online platform to assess whether there was a circadian rhythm in PO activity by analyzing the periodicity and rhythmicity of the data (Hutchison et al., 2015; Zielinski et al., 2014). The rhythmicity test was performed using BD2 eJTK, and the analysis presets were eJTK Classic (period of 24 h) using a cut‐off range of *p* < .05. To estimate the period of our data, we implemented the MFourFit preset with linear detrending. MFourFit is a curve‐fitting method that assumes a single period and returns the best‐fitting waveform for each cycle (Edwards et al., 2010). Additionally, we used a Gaussian distribution with a log‐link function to fit a generalized linear model (GLM) to PO activity assuming linear effects of age (6, 7, 8, 9 days), time of day, and the interaction between the two.

### 2.2 Feeding rate/Exposure assay

Given the age‐specific differences we observed in the PO assay, we assessed circadian‐driven variation in infection outcomes by measuring the effects of exposure age and exposure time. We used standard methods to account for *D. dentifera*'s rapid generation times. First, all neonates used in experiments were collected within a 24‐h period to prevent any age‐based differences. Second, to reduce any potential maternal effects, experimental neonates were collected from the third clutch of a single genotype of *D. dentifera* originally collected in Southern Michigan (USA) and reared under standard laboratory conditions (55 individuals per 1 L, 15:9 light:dark photoperiod at 22°C) for at least 30 generations. We also took additional precautions to standardize potential variation in laboratory‐reared algal food (*A. falcatus*) by freezing aliquots (1 mgC/L) of *A. falcatus* collected from a steady‐state chemostat (for details see Hite et al., 2020). This food was used from birth through the joint feeding and exposure assay to ensure that individuals would not change their feeding behavior in response to different algae and to prevent any algal growth during the assays.

We conducted a joint feeding and exposure assay following Hite et al. (2020). Specifically, all animals were maintained individually in 15 ml of COMBO (Kilham et al., 1998) and fed 1 mgC/L of *A. falcatus* (as described above) every 2 days until the start of the experiment. Both COMBO and algal cultures were prepared using filtered tap water (PureLab Ultra, Evoqua Water Technologies). We measured individual feeding rates in 6, 7, 8, and 9‐day‐old *D. dentifera* (maintained under a 15:9 light:dark photoperiod at 22°C) for 9 h that encompassed their entire active phase (night, 10 p.m. – 7 a.m.) and the corresponding 9 h during the resting phase (day, 10 a.m. – 7 p.m.; *N* = 240; 30 individuals × 2 exposure times × 4 ages). Time since individuals were last fed does not change feeding rate measurements (see Appendix S1), so observed time of day differences in feeding rate, and exposure, are attributed to their diel feeding behavior (Pfenning‐Butterworth et al., 2021).

For the assay, individuals were isolated in 10‐ml tubes containing 1 mgC/L of algae and 300 spores/ml for 9 h (tubes were placed on a rotator to ensure that algae and spores did not settle to the bottom of the tube). At the end of 9 h, individuals were moved to new tubes containing only COMBO. Individual feeding rates were determined by calculating the difference in fluorescence between the *D. dentifera* experimental tubes and control tubes that contained only algae as (Hite et al., 2020; Sarnelle & Wilson, 2008):

$$\begin{array}{c} \text{Feeding rate}=\ln\;{\left(F_{\text{control}}/F_{D.\;\text{dentifera}}\right)}^{*}v/t, \end{array}$$

where *F*_control_ is the average fluorescence of control wells, *F*_*D. dentifera*_ is the fluorescence of an animal well, *v* is the volume of COMBO and algae in ml, and *t* is the time *D. dentifera* fed in hours (Sarnelle & Wilson, 2008). After the feeding assay, individuals were maintained in 15‐ml tubes for 12 days to track infection success. We moved individuals to fresh tubes containing 1 mgC/L of algae every 2 days. Twelve days after exposure, infections were diagnosed visually (following Ebert, 2005) and confirmed by counting spore density for each individual on a hemocytometer.

To assess whether *D. dentifera* have a daily rhythm in infection risk, we fit a logistic regression model to the infection outcome data assuming linear effects of age at exposure (6, 7, 8, 9 days), feeding rate, and time of exposure (day or night) as explanatory variables (R v.4.1.1, R Core Team, 2021). We used likelihood ratio tests to assess the significance of model terms and computed likelihood ratio confidence intervals for the odds of infection when exposed during night versus day (*car* package, Fox & Weisberg, 2018; *mcprofile* package, Gerhard, 2016). If there is a daily rhythm in infection risk, we would expect time of exposure to have a biologically meaningful effect on probability of infection. We included age at exposure as a predictor to determine whether differences in body size or immunity had an effect on infection risk. We included feeding rate at exposure as a predictor to determine whether differences in feeding behavior effected infection risk.

To assess whether the intensity of infection (parasite fitness) depends on whether *D. dentifera* were exposed during their resting or active phase, we fit zero‐inflated regression models to the spore count data. Zero‐inflated count models allow us to simultaneously model the probability of zero‐spore count, which can occur from noninfection or clearance of infection, and the expected spore count. For the zero‐inflation component of the model, we included explanatory variables that were significant in the logistic regression model. To model the spore counts, we fit a zero‐inflated negative binomial model (ZINB), since these are appropriate for modeling counts where the variance is greater than the mean, which is common in ecological data. We used a log link function which is a standard link function for a generalized linear model when the parameter of interest (the mean) can only take on positive real values. In the linear predictor of both the zero‐inflated negative binomial models, we included the linear effect of age at exposure (6, 7, 8, and 9 days), feeding rate at exposure, time of exposure (resting/day or active/night), and the interaction between them as explanatory variables for the linear predictor. We then performed Wald tests to obtain inferences for the difference in mean spore count between active and resting phase exposure (*emmeans* package, Lenth et al., 2018). We used zero‐inflated models to estimate the mean difference in spore count for both individuals that were exposed conditional on becoming infected as well as individuals that were exposed regardless of their infection outcome (*countreg* package, Kleiber & Zeileis, 2016).

## 3 RESULTS

### 3.1 Phenoloxidase assay

**FIGURE 2:**
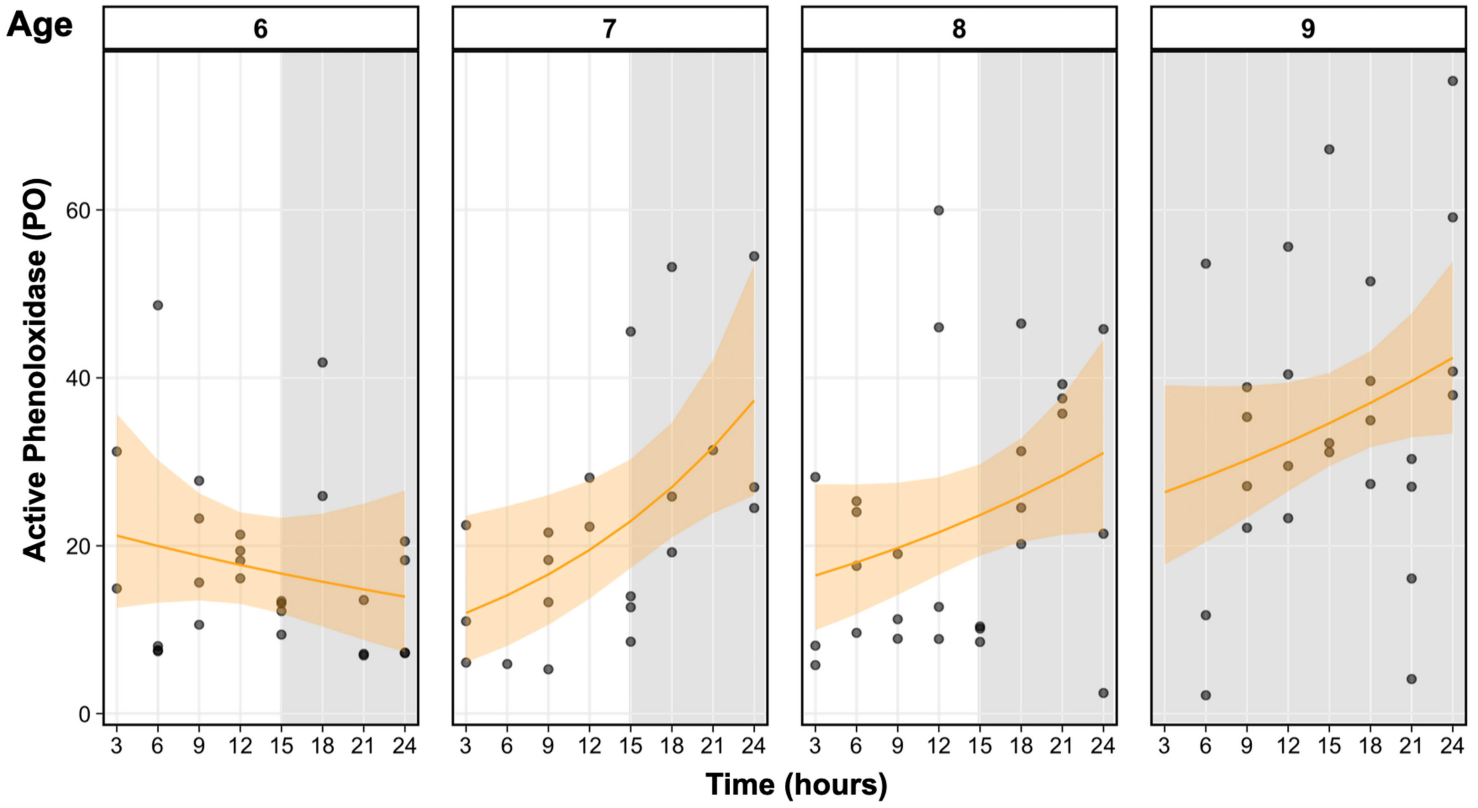
Predicted active phenoloxidase (PO) every 3 h over a 24‐h period for eight individuals pooled by age (6–9 days old, *n* = 4). Plot shading indicates the light–dark cycle, with 6–8‐day‐old individuals under a 15:9 light–dark cycle and 9‐day‐old individuals under 24‐h darkness. Points indicate individual active PO samples, lines are the predicted active phenoloxidase from a generalized linear model fit per age, time (hours), and their interaction, and shading denotes 95% Wald confidence intervals.

We found no evidence for a circadian rhythm in active phenoloxidase (PO) in *D. dentifera* (Figure 2). All samples were false for 24‐h rhythmicity at a threshold of *p* < .05, and the period was estimated at 29.25 ± 2.75 h. Instead, we saw that PO increased linearly with time for 7‐day‐olds only (GLM, *p* = .02, with an *R* ^2^ = .29) and this relationship was consistent when we corrected for body size (GLM, *p* = .01, with an *R* ^2^ = .21).

### 3.2 Infection probability

**FIGURE 3:**
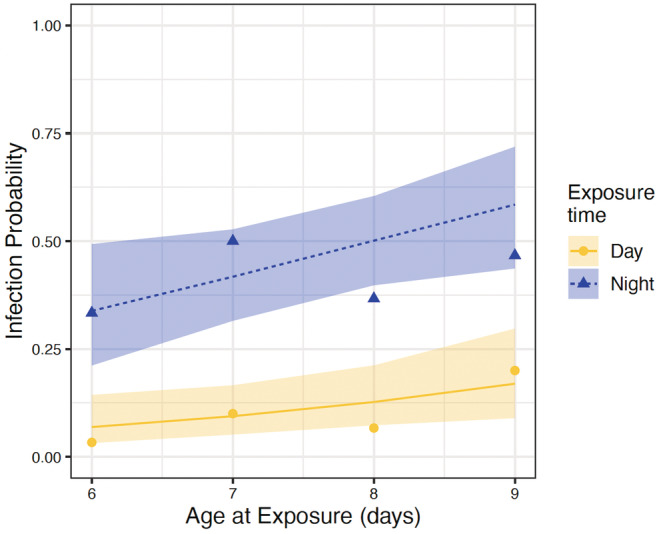
Predicted probability of infection (lines) and observed proportion (points) of exposed *Daphnia dentifera* during resting/day (yellow, solid, circles) or active/night (blue, dotted, triangles) that were infected. Shaded regions denote 95% Wald confidence intervals for the mean probability of infection. Data for the observed and predicted probabilities are given in Appendix S1: Table S1.

We found no evidence for the effect of feeding rate on infection probability (deviance: −2 log[Λ] = 0.65, *p* = .42). However, we found strong evidence for an effect of time of exposure (−2 log[Λ] = 31.34, *p* = 2.16 × 10^−8^) and evidence for an effect of age at exposure (−2 log[Λ] = 4.60, *p* = .032) on infection probability (Figure 3). The odds of infection were increased 590% (95% CI: 240–1401%) when *D. dentifera* were exposed during their active phase (night) compared with when they were exposed during their resting phase (day), when age and feeding rate at exposure were held constant. A 1‐day increase in age at exposure increased the odds of infection by 40% (3–94%) when time of exposure and feeding rate were held constant.

### 3.3 Infection intensity (spore count)

**FIGURE 4:**
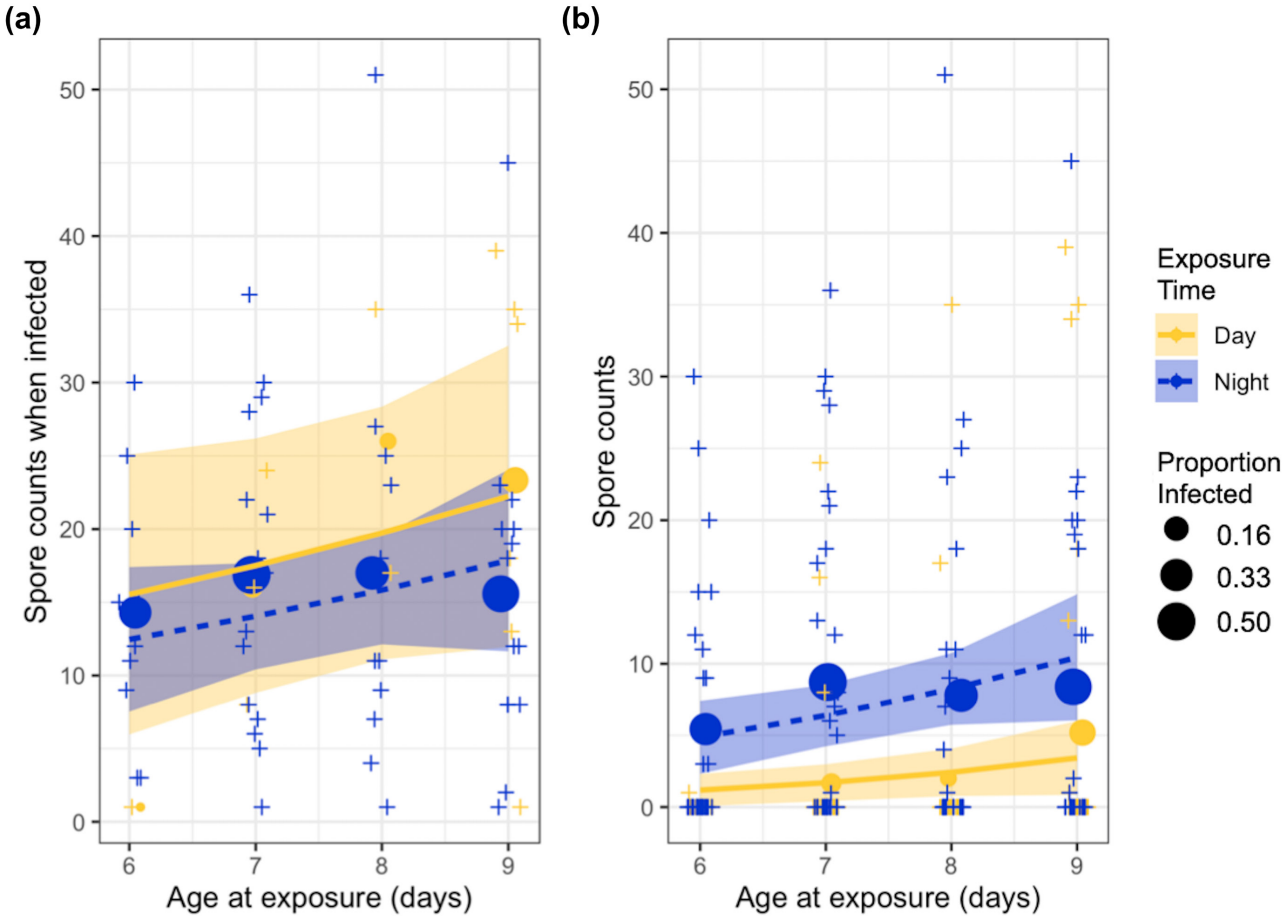
(a) Predicted mean spore counts conditioned on infection after exposure. (b) Predicted mean spore counts for exposed *Daphnia dentifera* regardless of infection outcome. Both plots include model predicted mean spore counts (lines) and observed mean spore count (circles, sized by the proportion of infected individuals) of *D. dentifera* exposed during resting/day (yellow, solid) or active/night (blue, dotted). Crosses are observed spore counts of individual exposed *D. dentifera*. The shaded regions denote 95% Wald confidence intervals for the expected spore count. Data for the observed and predicted probabilities are given in Appendix S1: Table S2.

The model included the phase at exposure and the linear effect of age for the zero‐inflation component of the model, since these terms were significant in the infection probability models described previously. We did not find statistical evidence that the average number of spores (infection intensity) differs between individuals who were infected during resting phase (day) versus active phase (night) exposures (active phase had 3.67 fewer spores than resting phase; 95% CI: 12.8 to −5.47; *p* = .43). However, when accounting for the increased probability of infection in individuals exposed during their active phase, we estimated that individuals exposed during their active phase (night) had a 5.24 (95% CI: 2.65–7.82; *p* < .0001) higher average spore count than individuals exposed during the resting phase (day) at the average age of exposure. In short, we found that the expected infection intensity of an individual does not depend on the phase of exposure if we know the individual is infected (Figure 4a). This suggests once an individual *Daphnia* becomes infected, they will have similar levels of disease regardless of the time of day they were exposed. However, if all we know is that the individual was exposed, we would expect individuals exposed during the active phase to have a higher expected infection intensity than individuals exposed during the resting phase, because they are more likely to become infected (Figure 4b).

## 4 DISCUSSION

Understanding how infection risk varies, whether among individuals, over ontogeny, or over the course of a day, requires disentangling the links between behaviors that mediate exposure and susceptibility. We assessed whether a known circadian rhythm in feeding behavior would lead to a rhythm in infection risk. We measured active PO levels over a circadian cycle, exposed individuals either during their active or during their resting phase, and quantified infection prevalence and within‐host infection intensity. We found that *D. dentifera* does not appear to have a circadian rhythm in one arm of the innate immune system (melanization pathway). However, there were time of day differences in infection risk between animals exposed during their active versus resting phase, indicating that individuals are more likely to become infected during the active phase (night).

Given that previous studies of immune gene expression show that several genes involved in the activation pathway for PO have a rhythm, with higher expression during the day (Rund et al., 2016), our finding that PO levels did not vary over a diel cycle was surprising. It is also somewhat surprising that previously documented rhythms in immune gene precursors are opposite of the *D. dentifera* circadian feeding and migration rhythms that indicate a clear rest/active cycle (Haney & Hall, 1975; Pfenning‐Butterworth et al., 2021), since many organisms upregulate immune pathways that are important for fighting disease exposure during their active phase (Curtis et al., 2014; Gibbs et al., 2012; Scheiermann et al., 2012, 2013). This could indicate that precursor genes are expressed out of phase because the immune pathway takes time and the active immune component is expressed hours later, potentially during the active phase of *Daphnia*.

Active PO did not have a daily cycle, but rather remained relatively constant across a day. The lack of a rhythm may arise because of resource intake on immunity—if the feeding rhythm created an offsetting circadian rhythm to immune gene expression. However, further work quantifying both immune phenotypes and immune gene expression over a diel cycle would be necessary to tease apart these relationships. Moreover, the individuals used in the immune assay were not exposed to an immune challenge (pathogen exposure), because we wanted to see if there was rhythmicity in the ability to mount an immune response over the course of a day. Thus, we measured whether there is a circadian rhythm in constitutive immunity, rather than induced immunity. Future studies could determine whether there are times of day when induced immunity is high and low by quantifying immune responses in immune‐challenged and unchallenged individuals across a daily cycle. These types of studies would further our understanding of circadian rhythms in immune function in response to disease.

Since *Daphnia* did not show a rhythm in PO levels, we predicted they would have a rhythm in infection risk that corresponds with their circadian rhythm in feeding (Figure 1f). Our results corroborate this prediction because individuals exposed during their active phase (night) had a significantly higher infection prevalence than those exposed during the resting phase (day). This suggests that the circadian rhythm in feeding behavior drives a circadian rhythm in infection risk. This result is not surprising given that increased feeding should also increase exposure to the parasite in this experiment. We chose to focus on PO as our measure of immunity because it plays a key role in the invertebrate immune system (Cerenius & Söderhäll, 2004; González‐Santoyo & Córdoba‐Aguilar, 2012; Povey et al., 2014); is activated by many invertebrate pathogens (Labbe & Little, 2009; Pauwels et al., 2010); and immune genes involved in its activation are rhythmic (Rund et al., 2016). It is of course possible that, had we measured other immune phenotypes, we could have found immune rhythms that are in phase with the feeding rhythm (Figure 1c). For instance, recent work demonstrates that hemocytes play a key role in fighting *M. bicuspidata* infection in *D. dentifera* (Stewart Merrill & Cáceres, 2018), and work in insect systems reveals higher hemocyte activity at night (Islam & Roy, 1982; Stone et al., 2012). Regardless, even if opposing immune rhythms exist for other arms of the *D. dentifera* immune system, they were clearly insufficient to override the rhythm in exposure caused by feeding.

While infection prevalence was significantly different between active and resting phase exposures, infection intensity was only significantly different between active and resting phase exposure when accounting for all exposed individuals, not just the individuals that become infected. This suggests that feeding behavior at the time of exposure contributes to infection success, while other factors are likely to mediate within‐host–pathogen intensity. For example, environmental stress can cause decreased *M. bicuspidata* intensity in *Daphnia* (e.g., copper contamination, Civitello et al., 2012; diet, Manzi et al., 2020). Additionally, host‐specific traits such as age and body size can drive differences in infection intensity (Graham, 2003; Woolhouse, 1998). Immune response is often positively correlated with infection intensity (Schultz et al., 2018), which highlights the general principle that a stronger immune response does not necessarily translate into a healthier individual (see Graham et al., 2011).

Our results show that the time of day that individuals are exposed to pathogens affects the likelihood of infection. Numerous studies have indicated that infection during an organism's resting phase can have drastic consequences for the host's survival and immune response, as well as pathogen fitness (see Hopwood et al., 2018; Westwood et al., 2019). For example, *Salmonella* colonization and host inflammatory responses were higher in mice infected during the resting phase than those infected during the active phase (Bellet et al., 2013). These studies suggest that infection outcomes are often more severe when hosts are infected during the phase opposite of when they would be exposed to pathogens in nature (typically the active phase) and these differences have important implications for the conclusions drawn from epidemiological studies.

Here, however, we observe the opposite pattern: Infection risk is higher when animals are exposed during their active phase because their activity—feeding—increases their exposure. This suggests the potential for an interaction between the other major circadian rhythms in *Daphnia* diel vertical migration. The effects of the observed circadian rhythm in infection risk on population‐level processes (i.e., size of epidemics) will depend on the distribution of parasites through the water column (Overholt et al., 2012; Shaw, 2019). If pathogens occur in surface waters where *Daphnia* spend their active phase, then the circadian rhythm in exposure and infection risk should lead to large epidemics, whereas if pathogens occur in deeper waters where *Daphnia* spend their resting phase, then epidemics should be smaller.

This is the first study, to our knowledge, that demonstrates a circadian variation in infection risk in *Daphnia*. More generally, our results indicate that the natural circadian rhythm of the host, and parasite, should be taken into consideration when designing experiments and models of epidemiology. Especially in the laboratory, infections are typically carried out during the day, which may lead to a misleading understanding of infection risk in a natural setting. For example, daytime pathogen exposure for a nocturnal host may enhance or reduce infection outcomes, depending on the interaction between exposure and susceptibility.

## AUTHOR CONTRIBUTIONS

**Alaina C. Pfenning‐Butterworth:** Conceptualization (lead); data curation (lead); formal analysis (supporting); writing – original draft (lead); writing – review and editing (lead). **David T. Nguyen:** Formal analysis (lead); writing – original draft (supporting); writing – review and editing (supporting). **Jessica L. Hite:** Conceptualization (supporting); data curation (supporting); writing – original draft (supporting). **Clayton E. Cressler:** Conceptualization (supporting); writing – original draft (supporting); writing – review and editing (supporting).

## CONFLICT OF INTEREST

The authors have no potential conflict of interest with respect to the research, authorship, and/or publication of this article.

## Supporting information


Appendix S1

## Data Availability

Code to fully replicate this study is available at: https://github.com/alainapb/Infection_risk_rhythm. Data are available at: https://doi.org/10.5061/dryad.c866t1g9j.
