# Supplementary material for: Circadian rhythms mediate infection risk in Daphnia dentifera
Source: Ecol Evol. 2022 Sep 9;12(9):e9264. doi: 10.1002/ece3.9264 (PMC9463024; doi:10.1002/ece3.9264)
Supplement: Supplementary file 1 — Appendix S1 [file ECE3-12-e9264-s001.docx]

**Appendix S1**


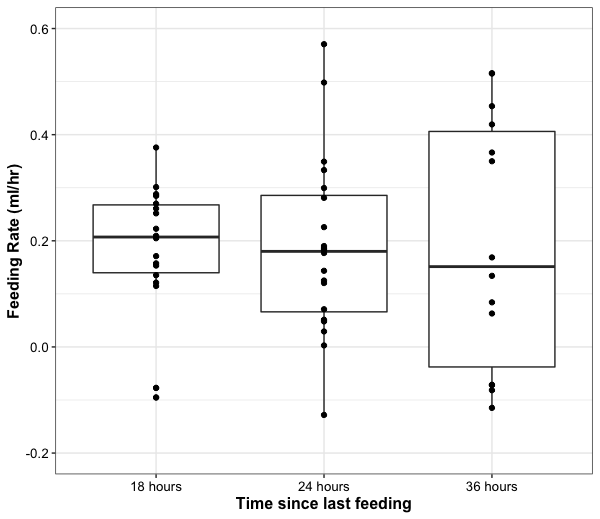


**Appendix S1: Figure S1.** Median feeding rate (ml/hr) for 6-day-old individuals that differ in the hours since they were last fed. Points indicate individual feeding rates. There is no significant difference in feeding rates between different lengths of time since individuals were last fed (ANOVA, F_2,54_ = 1.99, *p* = 0.146).

**Appendix S1: Table S1**. Observed infection prevalence (no. of infected individuals / no. of exposed individuals) and the estimated infection prevalence (Wald 95% confidence intervals) from the logistic regression model.

| **Age** | **Time treatment** | **Observed Infection Prevalence** | **Estimated Infection Prevalence** |
| --- | --- | --- | --- |
| 6 | Day | 0.03 (1/30) | 0.03 (0.03, 0.14) |
| 6 | Night | 0.33 (10/30) | 0.33 (0.21, 0.49) |
| 7 | Day | 0.1 (3/30) | 0.1 (0.05, 0.17) |
| 7 | Night | 0.5 (15/30) | 0.5 (0.32, 0.53) |
| 8 | Day | 0.07 (2/30) | 0.07 (0.07, 0.21) |
| 8 | Night | 0.37 (11/30) | 0.37 (0.4, 0.6) |
| 9 | Day | 0.2 (6/30) | 0.2 (0.09, 0.3) |
| 9 | Night | 0.47 (14/30) | 0.47 (0.44, 0.72) |

**Appendix S1: Table S2.** Mean of the observed spore counts (variance), predicted mean spore counts regardless of infection outcome, predicted mean spore counts conditioned on infection after exposure, the number of individuals infected and the mean feeding rate for each age and exposure time treatment.

| **Age** | **Time treatment** | **Observed mean spore counts** | **Predicted mean spore counts** | **Conditional predicted mean spore counts** | **No. of infected individuals** | **Mean feeding rate (ml/hr)** |
| --- | --- | --- | --- | --- | --- | --- |
| 6 | Day | 0.04 (0.04) | 1.20 | 15.68 | 1 | 0.34 |
| 6 | Night | 5.43 (73.88) | 4.91 | 12.59 | 10 | 0.07 |
| 7 | Day | 1.66 (29.16) | 1.71 | 17.68 | 3 | 0.08 |
| 7 | Night | 8.72 (129.99) | 6.45 | 14.19 | 15 | -0.21 |
| 8 | Day | 2.00 (56.40) | 2.44 | 19.93 | 2 | 0.01 |
| 8 | Night | 7.79 (162.61) | 8.32 | 16.00 | 11 | 0.23 |
| 9 | Day | 5.19 (141.16) | 3.46 | 22.46 | 6 | 0.39 |
| 9 | Night | 8.38 (127.21) | 10.55 | 18.03 | 14 | 0.19 |
